# GnRH agonist versus GnRH antagonist in assisted reproduction cycles: oocyte morphology

**DOI:** 10.1186/1477-7827-10-33

**Published:** 2012-04-27

**Authors:** Ana Marcia M Cota, Joao Batista A Oliveira, Claudia G Petersen, Ana L Mauri, Fabiana C Massaro, Liliane FI Silva, Andreia Nicoletti, Mario Cavagna, Ricardo LR Baruffi, José G Franco

**Affiliations:** 1 Department of Gynecology and Obstetrics, Botucatu Medical School, São Paulo State University—UNESP, Botucatu, Brazil; 2 Center for Human Reproduction Prof. Franco Junior, Ribeirao Preto, Brazil; 3 Paulista Center for Diagnosis, Research and Training, Ribeirao Preto, Brazil; 4 Women’s Health Reference Center, Perola Byington Hospital, Sao Paulo, Brazil

**Keywords:** Oocyte morphology, *in vitro* fertilization, Gonadotropin-releasing hormone analogues, Assisted reproduction technique

## Abstract

**Background:**

The selection of developmentally competent human gametes may increase the efficiency of assisted reproduction. Spermatozoa and oocytes are usually assessed according to morphological criteria. Oocyte morphology can be affected by the age, genetic characteristics, and factors related to controlled ovarian stimulation. However, there is a lack of evidence in the literature concerning the effect of gonadotropin-releasing hormone (GnRH) analogues, either agonists or antagonists, on oocyte morphology. The aim of this randomized study was to investigate whether the prevalence of oocyte dysmorphism is influenced by the type of pituitary suppression used in ovarian stimulation.

**Methods:**

A total of 64 patients in the first intracytoplasmic sperm injection (ICSI) cycle were prospectively randomized to receive treatment with either a GnRH agonist with a long-term protocol (n: 32) or a GnRH antagonist with a multi-dose protocol (n: 32). Before being subjected to ICSI, the oocytes at metaphase II from both groups were morphologically analyzed under an inverted light microscope at 400x magnification. The oocytes were classified as follows: normal or with cytoplasmic dysmorphism, extracytoplasmic dysmorphism, or both. The number of dysmorphic oocytes per total number of oocytes was analyzed.

**Results:**

Out of a total of 681 oocytes, 189 (27.8 %) were morphologically normal, 220 (32.3 %) showed cytoplasmic dysmorphism, 124 (18.2%) showed extracytoplasmic alterations, and 148 (21.7%) exhibited both types of dysmorphism. No significant difference in oocyte dysmorphism was observed between the agonist- and antagonist-treated groups (*P* ≫ 0.05). Analysis for each dysmorphism revealed that the most common conditions were alterations in polar body shape (31.3%) and the presence of diffuse cytoplasmic granulations (22.8%), refractile bodies (18.5%) and central cytoplasmic granulations (13.6%). There was no significant difference among individual oocyte dysmorphisms in the agonist- and antagonist-treated groups (*P* ≫ 0.05).

**Conclusions:**

Our randomized data indicate that in terms of the quality of oocyte morphology, there is no difference between the antagonist multi-dose protocol and the long-term agonist protocol. If a GnRH analogue used for pituitary suppression in IVF cycles influences the prevalence of oocyte dysmorphisms, there does not appear to be a difference between the use of an agonist as opposed to an antagonist.

## Background

In assisted reproduction, the selection of sperm, oocytes, and embryos to achieve better clinical results are important tasks for the embryologist. This goal has particular relevance when religious, ethical, or legal considerations limit embryo selection after fertilization. The morphological evaluation of oocytes, although subjective, is still the standard criterion to help identify the cells with the greatest potential for development. The denudation step not only allows for the evaluation of oocyte maturity by identifying the first polar body but also allows for morphological assessment of the zona pellucida, perivitelline space, and cytoplasm [1]. Oocyte dysmorphisms may occur due to age, genetic changes, and factors related to the treatment itself, including the ovarian stimulation and the hormonal environment to which the oocyte is exposed [2-4].

The first therapeutic uses of *in vitro* fertilization (IVF) were performed in natural cycles without ovarian stimulation. However, there is currently a consensus that better results are obtained with the induction of ovulation. Thus, different protocols are being developed, many of which rely primarily on the administration of gonadotropins to induce multifollicular development, thus increasing the number of available oocytes and thereby the number of developing embryos to be selected and transferred. However, the induction of ovulation might induce a premature surge in luteinizing hormone (LH), which would cause premature luteinization or ovulation and thereby prevent the collection of oocytes [5-9].

Both agonist and antagonist analogues of the gonadotropin-releasing hormone (GnRH) play an important role in reducing the incidence of premature LH surges by reversibly blocking the secretion of pituitary gonadotropins. It is estimated that without the use of GnRH analogues, a surge in LH occurs after approximately 20% of all IVF cycles/intracytoplasmic sperm injections (ICSI) [10,11]. As a result, the frequency of cancelled assisted conception cycles has decreased and pregnancy rates have increased. GnRH agonists (GnRH-a) have been used in assisted reproduction treatments since the 1980s. They suppress the release of gonadotropins, including follicle-stimulating hormone (FSH) and LH, by desensitizing pituitary receptors, a phenomenon called “down-regulation” [8,12]. GnRH antagonists (GnRH-ant) have only recently (since the late 1990s) been used as part of the therapeutic arsenal in the field of assisted reproduction [13]. They act by directly binding the GnRH receptors and block them in a competitive manner [8]. Thus, GnRH-ant cause an immediate, reversible, and rapid suppression of gonadotropin release [6,8,12,14].

Although it is accompanied by some disadvantages, GnRH-a have become well accepted in clinical practice, and their use is associated with an increase in the rate of pregnancy [15]. The development of GnRH-ant capable of blocking the pituitary receptors offered a new therapeutic option. Comparative studies between the two analogues have suggested that the use of antagonists is associated with a shorter duration of the ovulatory stimulus and a decreased incidence of ovarian hyperstimulation syndrome; however, the rates of pregnancy and live birth do not appear to be significantly affected, depending on the type of GnRH analogue used [15-20]. Takahashi et al. [21] found that the use of a GnRH-ant in controlled ovarian hyperstimulation improves the outcome of pregnancy in patients who have experienced several failed IVF/ICSI cycles under a GnRH-a protocol, most likely due to an improvement in the quality of the blastocysts generated. However, GnRH-a has applications in assisted reproductive technology cycles other than the down-regulation of pituitary receptors [15,22,23].

Although the human ovary expresses GnRH receptors, the mechanism of the action of GnRH on the ovary remains controversial and is not completely understood. Thus, when GnRH analogues are used in IVF cycles, their mechanism of action on the oocytes is unknown. GnRH receptors are found in the luteal cells and granulosa cells of the antral follicles but not in primordial and pre-antral follicles [24,25]. The correlation between the expression of GnRH receptors and the stage of follicular development suggests a direct influence of GnRH on folliculogenesis and oocyte development. Unfortunately, there are only a few studies on the possible action of GnRH analogues on oocyte phenotype [1,4,26]. Those results that have been published are often contradictory.

To better understand the effects of GnRH analogues on the ovaries, the objective of this prospective, randomized study was to evaluate whether the prevalence of oocyte dysmorphism is influenced by the type of pituitary suppression (GnRH-a or GnRH-ant) used in ovarian stimulation.

## Methods

### Participants

This prospective study was conducted on 64 women during the first ICSI cycle at the Center for Human Reproduction Professor Franco Junior. The inclusion criteria were as follows: age ≤37 years, first IVF/ICSI cycle, BMI ≪30 kg/m^2^, regular menses, and both ovaries present. The exclusion criteria were as follows: polycystic ovarian syndrome, severe endometriosis, ovarian cysts as assessed by transvaginal ultrasound, and basal FSH ≥10 IU/ml. Written consent was obtained from all the patients, and the study was performed according to the norms of the institutional ethics committee.

### Randomization

A double randomization process was used. First, a computer-generated table (first randomization) indicated which ovulation induction protocol (GnRH-a or GnRH-ant) was assigned. Then, lots were drawn (second randomization) to determine to which case that specific patient would correspond (i.e., case #1 or case #30). One nurse, blinded to subject identities, performed all the lot draws. In this manner, each patient was randomly allocated to one of two groups:

1. GnRH-a group (n = 32): long-term GnRH agonist (leuprolide acetate, Lupron®, Abbott, Brazil) protocol

2. GnRH-ant group (n = 32): multi-dose GnRH antagonist (cetrorelix, Cetrotide®, Serono, Brazil) protocol

### Ovarian stimulation

#### GnRH-a protocol

Pituitary down-regulation began during the luteal phase of the previous menstrual cycle with the GnRH-a leuprolide acetate (Lupron®) at a dose of 1 mg/day for 14 days. The ovaries were then stimulated with a fixed dose of 150 to 225 IU recombinant FSH (rFSH; Gonal F®; Serono) and 75 IU/day recombinant LH (rLH; Luveris®; Serono) for a period of 7 days. On day 8 of ovarian stimulation, follicular development was monitored by transvaginal ultrasound at 7 MHz (Medison Digital Color MT; Medison Co. Ltd., Seoul, Korea). The rFSH dose was adapted according to the ovarian response, and rLH supplementation was increased to 150 IU/day when one or more follicles measuring ≥10 mm in diameter were found.

#### GnRH-ant protocol

On Day 3 of the cycle, ovarian stimulation was induced with a fixed dose of 150 to 225 IU rFSH and 75 IU/day rLH for a period of 5 days. On day 8 of the menstrual cycle (day 6 of ovarian stimulation), follicular development was monitored by transvaginal ultrasound at 7 MHz. The rFSH dose was adapted according to the ovarian response, and rLH supplementation was increased to 150 IU/day when one or more follicles measuring ≥10 mm in diameter were found. The GnRH-ant cetrorelix (Cetrotide®) was started at a dose of 0.25 mg/day s.c. when at least one follicle of ≥14 mm was observed by ultrasound.

To induce the final oocyte maturation in both groups (GnRH-a and GnRH-ant), 250 μg of recombinant hCG (r-hCG; Ovidrel®, Serono) was administered s.c. when at least two follicles reached a mean diameter of ≥17 mm. GnRH-a and GnRH-ant were administered until the day of the hCG injection. Oocyte retrieval was performed by transvaginal aspiration under ultrasound guidance 34 to 36 hours following the r-hCG injection.

### Preparation of oocytes

The retrieved oocytes were incubated in culture medium (P1; Irvine Scientific, Santa Ana, CA, USA) at 37°C and 5.5% CO_2_ for 1 hour. Cumulus cells were removed by exposing the oocytes to modified human tubal fluid medium (mHTF; Irvine Scientific) containing 40 IU/ml hyaluronidase (Irvine Scientific) for 30 sec, after which coronal cells were manually removed using a stripper buffer (Cook, Australia). The denuded oocytes were classified according to their level of maturation. Oocytes with the first polar body, i.e., at the metaphase II (MII) stage, were considered to be mature and were used for the ICSI procedure.

### Oocyte morphology

**Figure 1:**
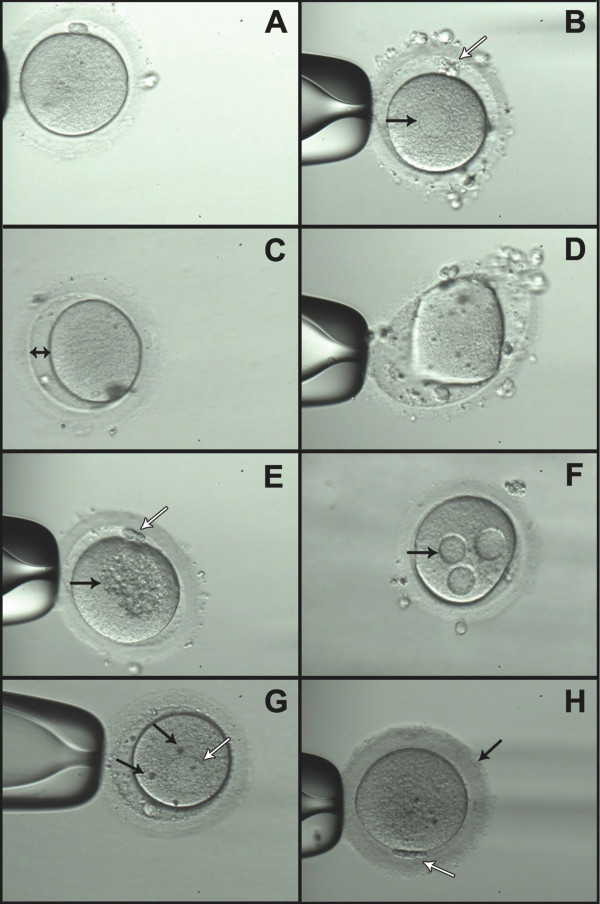
**Oocytes with normal and abnormal morphology: (A)** normal oocyte, **(B)** black arrow: smooth endoplasmic reticulum (SER) aggregations; white arrow: abnormal polar body **(C)** large perivitelline space **(D)** abnormal oocyte shape **(E)** black arrow: central cytoplasmic granulation; white arrow: abnormal polar body **(F)** cytoplasmic vacuoles **(G)** black arrow: refractile bodies; white arrow: smooth endoplasmic reticulum (SER) aggregations **(H)** black arrow: dark thick zona pellucida and cytoplasm; white arrow: abnormal polar body.

Figure 1 shows normal oocyte and oocytes with cytoplasmic and extracytoplasmic dysmorphisms.

Two to four hours after the retrieval and before being subjected to ICSI, MII oocytes from both groups were morphologically analyzed under an inverted light microscope (Nikon Eclipse TE 300 inverted microscope) by a single operator blinded to subject identities. The oocytes were classified as follows: [1,26-28].

Normal: clear, homogenous cytoplasm with a uniform texture, a round or ovoid first polar body (PB) with a smooth surface, and a zona pellucida and perivitelline space of normal size

Cytoplasmic dysmorphism: the presence of vacuoles, refractile bodies, granularity, and smooth endoplasmic reticulum (SER) aggregations

Extracytoplasmic dysmorphism: alterations of the zona pellucida (thick and/or dark), perivitelline space (large), polar body (fragmented, large, and/or degenerated), and oocyte shape (irregular).

Both cytoplasmic and extracytoplasmic dysmorphisms.

### Statistical analysis

The statistical analysis was performed using software R, version 2.13.0. The primary outcomes were the frequency of oocyte dysmorphism in each group (agonist X antagonist). First, statistical analysis was performed to verify the homogeneity of the variables at the individual level (age, body mass index (BMI), FSH) between the GnRH-a and GnRH-ant groups. Then, the differences between the groups at the level of the oocyte (presence or absence of dysmorphism) were analyzed. The Mann–Whitney test was used to verify the homogeneity of the non-normal quantitative variables; Student’s *t*-test was used for the normal quantitative variables. The chi-squared test was used for the qualitative variables. To determine whether either group had a significant effect on the proportion of oocytes with dysmorphisms or if a particular dysmorphism was more prevalent in any of the groups, a logistic regression model based on the generalized estimating equations (GEE) method was used.

The variables describing morphological oocyte features were presented as absolute and proportional frequencies. The data from the logistic regression analyses were presented as the odds ratio (OR) and 95% confidence interval (CI). The significance level was set at *P ≪* 0.05.

The sample size was calculated by a virtual comparison between two proportions. Data from the literature show that at least 20% of the MII oocytes have normal morphology [29-31]**.** Thus, a sample size of 300 oocytes in each group confers 80% power to detect an increase of 10% in one of the groups with an alpha significance level of 0.05 (two-tailed).

## Results

Basic demographic characteristics, such as maternal age, BMI, duration of infertility, smoking, alcohol use, and infertility etiology, were not significantly different (*P* ≫ 0.05) between the GnRH-a and GnRH-ant patient groups. The distribution (*P* ≫ 0.05) of the main characteristics of the ovarian stimulation cycle observed for the GnRH-a and GnRH-ant groups was equal. These data are summarized in Table 1.

**Table 1:** The basic demographic characteristics of the patients and the cycle of ovarian stimulation

	**Agonist group**	**Antagonist group**	***P***
Patients (n)	32	32	
Age (years)	33.2 ± 3.0	32.5 ± 3.0	0.39
BMI (kg/m^2^)	23.5 ± 3.2	23.2 ± 3.0	0.48
Duration of infertility (years)	4.4 ± 3.0	4.1 ± 3.0	0.33
**Infertility**			0.11
Primary	56.2% (18/32)	78.1% (25/32)	
Secondary	43.8% (14/32)	21.9% (7/32)	
Tobacco use (%)	3.1% (1/32)	0 (0/32)	0.31
Regular alcohol use	12.5% (4/32)	3.1% (1/32)	0.35
**Etiology**			0.59
Male factor	50% (16/32)	53.1% (17/32)	
Idiopathic	34.4% (11/32)	21.9% (7/32)	
Tubal factor	9.3% (3/32)	18.7% (6/32)	
Male + Tuboperitoneal	6.3% (2/32)	6.3% (2/32)	
Total dose FSH (UI)	2185.5 ± 617	1877.3 ± 817	0.09
Total dose LH (UI)	1094.5 ± 258	1026.5 ± 385	0.87
Time of stimulation (days)	11 ± 1.8	10.4 ± 3.2	0.82
**Follicles (n) (hCG day)**			
Total (≥10 mm) ≥16 mm	15.9 ± 8.3 6.9 ± 3.1	17.7 ± 9.2 7.5 ± 3.6	0.49 0.47
≥18 mm	4.1 ± 2.1	4.1 ± 1.8	0.65
**Retrieved oocytes:**			
Total	12.5 ± 6.9	13.4 ± 7.0	0.57
Metaphase II stage	9.3 ± 5.9	9.8 ± 6.0	0.77
Metaphase I stage	1.3 ± 1.5	1.4 ± 1.4	0.22
Germinal vesicle stage	1.3 ± 2.5	0.9 ± 1.4	0.88
Implantation rate	29% (18/62)	27.4% (17/62)	1.00
Pregnancy rate	40.6% (13/32)	37.5% (12/32)	0.79

A total of 681 oocytes were obtained: 330 oocytes from the GnRH-a group and 351 from the GnRH-ant group. Of these, 189 (27.8%) were morphologically normal, 220 (32.3%) had cytoplasmic dysmorphisms, 124 (18.2%) had extracytoplasmic alterations, and 148 (21.7%) had both dysmorphisms. The oocyte dysmorphisms were not significantly different between the GnRH-a and GnRH-ant groups (*P* ≫ 0.05). Logistic regression analysis revealed that the probability of finding a normal oocyte in the GnRH-ant group was higher (OR:1.29, 95% CI: 0.76-2.22) than in the GnRH-a group. However, the probability of detecting an oocyte with cytoplasmic dysmorphism in the GnRH-ant group was similar (OR:1.05, 95% CI: 0.62-1.81) to the probability of detecting such an oocyte in the GnRH-a group. However, the probability of detecting an oocyte with extracytoplasmic dysmorphism or both dysmorphisms in the GnRH-ant group was lower (OR: 0.78, 95% CI: 0:38–1:59 and OR: 0.77. 95% CI: 0.38-1.54, respectively) than for the GnRH-a group. Again, these groups were not significantly different. These data are summarized in Table 2.

**Table 2:** Comparison of the overall prevalence of oocyte dysmorphisms in the GnRH agonist and GnRH antagonist groups

**Oocyte characteristics**	**Total** **n: 681**	**Group**		***P***	**Odds ratio (95% CI)**	
		**Agonist**	**Antagonist**			
		**n: 330**	**n: 351**			
**Oocytes:**				0.34	1.29 (0.75–2.22)	
Normal	27.8% (189)	25.8% (85)	29.6% (104)			
Dysmorphic	72.2% (492)	74.2% (245)	70.4% (247)			
Cytoplasmic dysmorphism	32.3% (220)	32.1% (106)	32.5% (114)	0.84	1.05 (0.61–1.80)	
Extracytoplasmic dysmorphism	18.2% (124)	19.4% (64)	17.1% (60)	0.48	0.78 (0.37–1.59)	
Cytoplasmic + extracytoplasmic dysmorphism	21.7% (148)	22.7% (75)	20.8% (73)	0.45	0.77(0.38–1.54)	

It is possible that the oocytes exhibited more than one change in morphology. When each dysmorphism was analyzed, it was observed that alterations in polar body shape (31.8%, 217/681) and the presence of diffuse cytoplasmic granulations (22.8%; 155/681), refractile bodies (18.5%; 126/681) and central cytoplasmic granulations (13.6%; 93/681) were the most common alterations. Other morphological abnormalities were present in less than 10% of the oocytes analyzed. Interestingly, cytoplasmic vacuoles (0.7%; 5/681) and changes in oocyte shape (1.9%; 13/681) were not very common. The individual incidences of oocyte dysmorphism obtained from the GnRH-a and GnRH-ant groups were not significantly different (*P* ≫0.05). These data are summarized in Table 3.

**Table 3:** Comparison of the prevalence of specific dysmorphisms in the GnRH agonist and GnRH antagonist groups

**Oocyte characteristics**	**Total** **n:681**	**Group**		***P***	**Odds ratio (95% CI)**
		**Agonist**	**Antagonist**		
		**n:330**	**n:351**		
Cytoplasmic dysmorphism (presence of)					
Diffuse granulation	22.8% (155)	25.2% (83)	20.5% (72)	0.17	0.95 (0.44–2.06)
Central cytoplasmic granulation	13.6% (93)	12.1% (40)	15.1% (53)	0.32	1.06 (0.48–2.44)
Refractile bodies	18.5% (126)	20.3% (67)	16.8% (59)	0.27	0.88 (0.32–2.43)
SER aggregations	2.3% (16)	1.8% (6)	2.8% (10)	0.45	1.41 (0.25–7.85)
Vacuoles	0.7% (5)	0.9% (3)	0.6% (2)	0.93	0.66 (0.07–6.37)
Extracytoplasmic (alterations)					
Polar body shape	31.3%(213)	30.9%(102)	31.6%(111)	0.90	0.78 (0.38–1.61)
Perivitelline space	6.8% (46)	8.2% (27)	5.4% (19)	0.19	0.66 (0.28–1.52)
Zona pellucida	3.4% (23)	4.8% (16)	2.0% (7)	0.06	0.40 (0.15–1.06)
Oocyte shape	1.9% (13)	1.1% (7)	1.7% (6)	0.78	0.79 (0.26–2.35)

* oocytes could present more than 1 dysmorphism.

## Discussion

Oocyte quality is a major determinant of embryo quality and the subsequent success of fertility treatment. Thus, to improve the outcomes of assisted reproduction procedures, it is important to identify non-invasive parameters to evaluate oocyte quality. Among these, the examination of egg phenotype using optical microscopy is the most commonly used method.

A normal MII oocyte has a round shape, a single intact polar body, a clear zona pellucida, a small perivitelline space, and a transparent and smooth cytoplasm without granules or inclusions [1,27,30,32,33]. However, most of the oocytes collected after ovarian stimulation from infertile patients or from some fertile donors have some type of dysmorphism [27,28,30,32-37]. Various studies have demonstrated the clinical importance of oocyte dysmorphism. Indeed, specific intra- and extracellular morphological changes in oocytes, such as granulations, cytoplasmic inclusions, and abnormalities of the polar body, zona pellucida, perivitelline space, and oocyte shape, have been shown to be related to fertilization, cleavage, embryo development, and clinical outcomes [1,26,29,30,33-47]. However, the results are controversial. Other studies have reported the prognostic value of oocyte morphology with respect to the IVF results, depending on the dysmorphism and results analyzed [27,39,46-51]. The variance in results can be explained by the use of different morphological criteria and/or a lack of standardization in the assessment of oocytes. Additionally, the factors involved in the induction of the morphological changes may have contributed to this variability.

For the oocyte to be capable of being fertilized by a sperm, it needs to go through a series of events that lead to both nuclear and cytoplasmic maturity. However, these events may occur independently [1,46,52]. After denudation, it is possible to verify the presence of the first polar body, which proves the maturity of the oocyte nucleus. However, the assessment of cytoplasmic maturation is not well established. Some authors correlate this cytoplasmic maturity with the lack of cytoplasmic inclusions. In other words, a mature oocyte is one without granulation, with vacuoles, and with uniform and clear cytoplasm [53]. Therefore, the presence of cytoplasmic inclusions could represent cytoplasmic immaturity, which would affect fertilization and embryonic development. Although immature oocytes can be fertilized, the subsequent course of embryonic development is abnormal [53]. Extra-cytoplasmic alterations may also be related to the maturity of the oocyte. An increase in the perivitelline space could be related to the premature exocytosis of cortical granules, suggesting a post-mature cytoplasm [54]. The presence of granulation in the perivitelline space is likely to be a physiologic phenomenon related to oocyte maturity, mainly because the incidence of this change was significantly more frequent in oocytes at stage MII (mature oocytes) as compared with immature oocytes. The control of meiotic resumption in mammalian oocytes depends on a network of extracellular and intracellular molecular interactions [55-58]. Understanding the regulatory role of such interactions in oocyte maturation is important for the analysis of oocyte phenotype. Some mechanisms that regulate GnRH or gonadotropin-regulated oocyte quality/maturity may regulate gamete morphology.

GnRH receptors are expressed in the human ovary, but the action of GnRH analogues on oocyte morphology and quality remains controversial. In the present study, no significant difference was observed between the incidence of oocyte dysmorphisms and the type of analogue used for pituitary suppression during IVF. In contrast to our findings, Murber *et al.*[4] found a significantly higher incidence of cytoplasmic changes in the GnRH-ant group than in the GnRH-a group (62.1% and 49.9%, respectively; *P* ≪ 0.01). In another study in which different stimulation protocols were evaluated, Otsuki *et al.*[26] found that SER aggregations were more common in short GnRH-a protocols as compared with long-term protocols. However, differences in the study design [4,26], in the populations, and in the analysis methods prevent comparisons with our findings. Our findings are in agreement with those of Rienzi *et al.*[1], who analyzed 1,191 MII oocytes. Their retrospective study included three distinct protocols for controlled ovarian hyperstimulation, a long-term GnRH-a protocol (268 cycles), a GnRH-ant protocol (142 cycles), and a natural cycle with minimal stimulation (106 cycles). No correlation was found between the protocol used for ovarian hyperstimulation and oocyte morphology [1].

Pharmacological doses of gonadotropins in stimulated cycles create a hormonal environment that induces the growth of a cohort of follicles that would otherwise degenerate under *in vivo* conditions. Thus, the type of gonadotropin used is also a factor that likely influences the quality of the oocyte. In our study, regardless of the analogue used (agonist or antagonist), all cycles were stimulated by r-LH in addition to r-FSH, and this factor has yet to be analyzed. Some studies have reported the beneficial effects of using r-LH on ovarian physiology and clinical outcomes [59,60]. In addition, Ruvolo *et al.*[61] suggested that supplementation with r-LH improves the chromatin quality of cumulus cells and protects them from apoptosis, possibly acting directly on granulosa cells or via a paracrine effect. These authors suggested that by maintaining the physiological function of the cumulus cells over time, the nuclear and cytoplasmic maturation of the oocyte would also be maintained, which would improve the quality of the oocytes at the time of retrieval. Detti *et al.*[62] speculated that supplementation with LH and FSH may have had a positive effect on oocyte quality. Acevedo *et al.*[63] randomly assigned 20 oocyte donors to ovarian stimulation protocols using GnRH-ant alone or GnRH-ant with recombinant LH and observed that LH activity supplementation improved oocyte quality. Thus, it can be hypothesized that the r-LH used in our study had a positive effect on oocyte morphology, which compensated for any possible deleterious effect of GnRH-a or GnRH-ant on ovarian physiology. In fact, Hernadez *et al.*[64] reported that a GnRH-ant inhibited the cell cycle by decreasing the synthesis of growth factors and thereby compromised the mitotic program of follicles and oocyte quality. However, this hypothesis needs to be tested with additional controlled trials. It should be noted that in our study, the overall incidences of oocyte dysmorphism in both the GnRH-a and GnRH-ant groups were similar to those reported in the literature, suggesting that any beneficial effects were associated with the protocols used for ovarian stimulation. In addition, Rashidi *et al.*[3] performed a randomized study comparing human menopausal gonadotropin (HMG) with r-FSH and did not find differences in the morphology of the MII oocytes retrieved.

Although there are several studies that have evaluated the clinical protocols of ovarian stimulation, very little is known regarding the correlation between these protocols and oocyte morphology. The majority of published studies that focus on oocyte morphology have examined its association with the results of IVF and not with the ovarian stimulation protocol [33,34,38,40,47,49]. In a recent meta-analysis [46], the oocyte dysmorphisms that were negatively correlated with fertilization rate, i.e., a lower chance of the fertilization of an oocyte, included changes in the first polar body, an increase in the perivitelline space, and the presence of refractile bodies and cytoplasmic vacuoles. The present study found that 72.2% of oocytes had at least one morphologic change, which is consistent with the 60-80% values reported by Figueira *et al*. [52] (60.2%); Rienzi *et al.*[1] and Yakin *et al*. [29] (84%); Kahraman *et al*. [39] (65.8%); Aguiar *et al*. [65] (67.3%); Balaban *et al*. [32] (63.1%); and De Sutter *et al*. [27] (63.5%). Among the dysmorphisms, cytoplasmic changes were the most common (32.3%), followed by the simultaneous occurrence of cytoplasmic and extracytoplasmic changes (21.7%). Strictly extracytoplasmic changes were the least common (18.2%). Conflicting data were reported by Balaban *et al.*[32], who showed a higher percentage of extracytoplasmic changes, followed by extra- and cytoplasmic changes and then finally by cytoplasmic changes. Mikkelsen and Lindenberg [54] also reported a higher incidence of extracytoplasmic changes followed by a smaller increase in the frequency of cytoplasmic changes.

When the dysmorphisms were evaluated individually, the most common cytoplasmic change found was cytoplasmic granularity (diffuse and central), followed by refractile bodies, SER aggregations and, finally, the presence of vacuoles. Balaban *et al.*[32] reported a different order of prevalence (in decreasing order of frequency): dark cytoplasm, refractile bodies, and granular cytoplasm. Rienzi *et al.*[1] reported an order of prevalence similar to ours: cytoplasmic granularity was the most common, followed by refractile bodies, vacuoles, and SER; the only difference was in the observed frequencies of vacuoles and SER. In our study, the most prevalent changes in the extracytoplasmic dysmorphisms were related to the first polar body, followed by changes in the perivitelline space, zona pellucida, and oocyte shape, as reported by Rienzi *et al.*[1]. However, Balaban *et al.*[32] showed that the most frequent extracytoplasmic alterations were an increase in the perivitelline space, followed by a dark pellucida area and shape abnormalities. Aguiar *et al.*[48] showed that 42.8% of oocytes had multiple changes, 40% had cytoplasmic inclusions, 14.3% exhibited fragmentation of the polar body, and 2.9% displayed enlargements of the perivitelline space.

Some limitations of our study should be noted. The population of 64 patients is small (32 patients per group), despite the inclusion of over 300 eggs per group. This low number may have influenced the results. However, a study [1] involving a larger number of patients did not reveal significant differences in oocyte morphology in relation to the ovarian stimulation protocol used. Our study is randomized in terms of the patient population, but the statistical analysis is calculated based on the number of eggs. If the patient population is randomized, it is fair to assume that the egg quality is similar up until stimulation and that any changes observed will be due to the stimulation itself. While randomization could compensate for any bias in terms of known and unknown patient-related factors that influence egg quality, certain factors will tend to be over-represented by those patients who provide more eggs (on average each patient provided 10 eggs). However, it should be noted that, again, a study [1] that limited the number of oocytes analyzed per patient did not reveal significant differences in oocyte morphology in relation to the ovarian stimulation protocol used.

## Conclusions

Our randomized data indicate that in terms of the quality of oocyte morphology, there is no difference related to the use of a multi-dose antagonist protocol or a long-term agonist protocol. If a GnRH analogue used for pituitary suppression in IVF cycles influences the prevalence of various oocyte dysmorphisms, there does not appear to be a difference between the use of an agonist or an antagonist. To the best of our knowledge, this is the first randomized trial that has analyzed this association. A future randomized controlled trial with a larger sample size would be helpful to corroborate these findings.

## Competing interest

The authors declare that they have no competing interest.

## Authors’ contributions

AMMC participated in the design of the study, data interpretation, and the drafting of the manuscript. All authors were responsible for the data collection, analysis, and interpretation presented in the manuscript. JBAO participated in the design of the study, collected the data, and coordinated the study. CGP conducted the laboratory analysis of oocytes. JGFJ participated in critical revision of the manuscript. All authors read and approved the final manuscript.
